# The Public Health Impact of Coccidioidomycosis in Arizona and California

**DOI:** 10.3390/ijerph8041150

**Published:** 2011-04-15

**Authors:** Richard F. Hector, George W. Rutherford, Clarisse A. Tsang, Laura M. Erhart, Orion McCotter, Shoana M. Anderson, Kenneth Komatsu, Farzaneh Tabnak, Duc J. Vugia, Ying Yang, John N. Galgiani

**Affiliations:** 1 Global Health Sciences, University of California, San Francisco (UCSF),1200 Beale St., #1200, San Francisco, CA 94105, USA; E-Mail: grutherford@psg.ucsf.edu (G.W.R.); 2 Arizona Department of Health Services, 150 N 18th Ave., Ste. 140, Phoenix, AZ 85007, USA; E-Mails: tsangc@azdhs.gov (C.A.T.); erhartl@azdhs.gov (L.M.E.); mccotto@azdhs.gov (O.M.); anderssm@azdhs.gov (S.M.A.); komatsk@azdhs.gov (K.K.); 3 Infectious Diseases Branch, Division of Communicable Disease Control (DCDC), Center for Infectious Diseases (CID), California Department of Public Health, P.O. Box 997377, Sacramento, CA 95899, USA; E-Mails: farzaneh.tabnak@cdph.ca.gov (F.T.); duc.vugia@cdph.ca.gov (D.J.V.); ying.yang@cdph.ca.gov (Y.Y.); 4 Valley Fever Center for Excellence, College of Medicine, The University of Arizona, 1656 E. Mabel Street, Tucson, AZ 85724, USA; E-Mail: spherule@email.arizona.edu (J.N.G.)

**Keywords:** coccidioidomycosis, community-acquired pneumonia, diagnostics, azoles, vaccine

## Abstract

The numbers of reported cases of coccidioidomycosis in Arizona and California have risen dramatically over the past decade, with a 97.8% and 91.1% increase in incidence rates from 2001 to 2006 in the two states, respectively. Of those cases with reported race/ethnicity information, Black/African Americans in Arizona and Hispanics and African/Americans in California experienced a disproportionately higher frequency of disease compared to other racial/ethnic groups. Lack of early diagnosis continues to be a problem, particularly in suspect community-acquired pneumonia, underscoring the need for more rapid and sensitive tests. Similarly, the inability of currently available therapeutics to reduce the duration and morbidity of this disease underscores the need for improved therapeutics and a preventive vaccine.

## 1. Introduction

Coccidioidomycosis (also known as “Valley Fever”), is a disease caused by *Coccidioides immitis* and *C. posadasii*, two nearly identical species of pathogenic fungi found only in the Western Hemisphere. *Coccidioides* spp. grow in the top 2–8 inches of soil in semi-arid parts of the southwest region of the United States, parts of northern Mexico, and Central and South America, and produces arthroconidia, highly infectious propagules that can be disrupted from the ground and inhaled, where they can produce acute pulmonary infection in humans and many mammals [1–3]. Sixty percent of people infected with the fungus will remain asymptomatic or present with mild respiratory symptoms [4]. Approximately 40% of infections result in symptomatic disease, typically arising one to four weeks after exposure, which can resemble ordinary influenza, with fever, cough, fatigue, dyspnea, headache, myalgia and arthalgia. The disease is considered non-transmissible, although rare instances of transmission have occurred, including via organ transplantation [5] and inhalation of spores growing in visecro-cutaneous fistulae. A low percentage of symptomatic infections progress, resulting in extrathoracic dissemination, with hematogenous spread to the central nervous system, skin, joints, major organs or bones and result in chronic (months to years) and even fatal disease [6,7]. Persons at increased risk of disseminated coccidioidomycosis include immunocompromised persons, e.g., HIV/AIDS, diabetics, pregnant women [8,9] and persons of certain race/ethnicities, particularly Blacks and Filipinos [10,11].

The purpose of this article is to present information on the current incidence of disease and selected demographic information on those afflicted in Arizona and California, the two states most impacted by coccidioidomycosis. In addition, this article presents information on the need for earlier diagnosis of disease and improved diagnostic tests, reviews current and future therapeutics for the treatment of this disease, and, lastly, the possibility of developing a preventive vaccine for coccidioidomycosis.

## 2. The Public Health Impact of Coccidioidomycosis in Arizona

Every year, an estimated 150,000 people in the United States become infected with *Coccidioides* spp., and approximately 50,000 develop symptoms [12]. In the United States, Arizona has the highest number of reported cases, accounting for 60% of all national cases [13].

The Arizona Department of Health Services (ADHS) continues to monitor coccidioidomycosis in order to determine optimal ways to control this disease and minimize its burden on the people of Arizona. In this section, we describe the epidemiology of reported coccidioidomycosis in Arizona, validate the coccidioidomycosis surveillance system, investigate the diagnosis of coccidioidomycosis in patients with community-acquired pneumonia, and assess the effects of coccidioidomycosis in Arizona.

### 2.1. Epidemiology of Coccidioidomycosis

**Figure 1.**
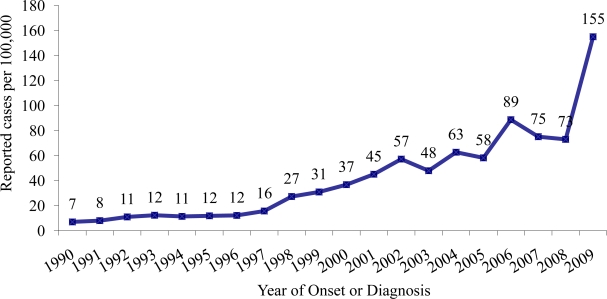
Rates of reported coccidioidomycosis cases in Arizona, 1990–2009.

Physicians in Arizona began reporting cases of coccidioidomycosis to the ADHS in the 1930s. Noticeable increases in reported coccidioidomycosis cases became apparent in the early 1990s. From 1990 through 1995, the number of annually reported cases more than doubled from 255 (7/100,000 population) to 623 (15/100,000 population) [14]. This increase led the ADHS to change its reporting rules to make coccidioidomycosis a laboratory-reportable disease in 1997. Since implementation of this mandatory requirement, reports of coccidioidomycosis have drastically increased in Arizona. In 2006, the number of cases peaked at 5,535 (89/100,000 population) and decreased to 4,815 (75/100,000 population) in 2007 and to 4,768 (73/100,000 population) in 2008. The increase in incidence of coccidioidomycosis could be due to a number of factors, including soil disturbance due to construction for Arizona’s rising population and an influx of susceptible individuals into the population [15,16]. In June 2009, a major commercial laboratory changed its reporting practice by beginning to report positive coccidioidomycosis enzyme immunoassay (EIA) results without confirmation by immunodiffusion assay in accordance with Arizona reporting rules, thereby further increasing the number of reported coccidioidomycosis cases. In 2009, Arizona reported 10,233 coccidioidomycosis cases (155/100,000 population) (see Figure 1).

**Figure 2.**
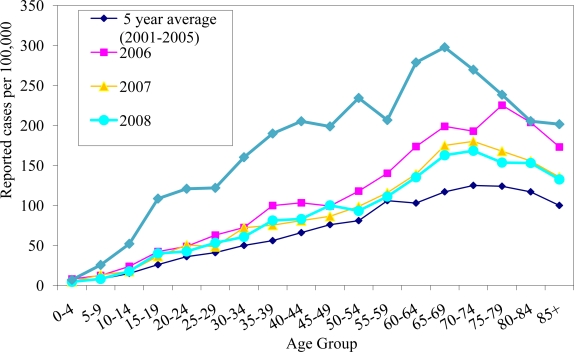
Rates of reported coccidioidomycosis cases by age group in Arizona, 2001–2009.

In 2006–2008, the median age of patients with coccidioidomycosis was 52 years (mean = 51). Since 2006, the median age has been decreasing. In 2006, the highest rate was in 75–79 year olds (225/100,000). In 2007 and 2008, the highest rates were in 70–74 year olds (180/100,000 and 168/100,000 respectively). Interestingly, in 2009, the median age of people with coccidioidomycosis was 47 years (mean = 47), while the highest rate was in 65–69 year olds (298/100,000) (Figure 2).

During 2006–2008, 54% of patients with coccidioidomycosis were male (84/100,000), and 46% were female (72/100,000). Of note, in 2009, the majority of cases were female (55%, 169/100,000) whereas only 45% (138/100,000) were male; a reversal of the earlier period.

Based on race and ethnicity data, the highest rates of coccidioidomycosis occurred in African Americans, with a rate of 49/100,000 from 2006–2008 and 67/100,000 in 2009. The rates in Asians or Pacific Islanders were 27/100,000 from 2006–2008 and 36/100,000 in 2009. In American Indian or Alaska Natives, the rates were 24/100,000 from 2006–2008 and 37/100,000 in 2009. White non-Hispanics had a rate of 17/100,000 from 2006–2008, and 28/100,000 in 2009 while Hispanics had a rate of 14/100,000 from 2006–2008, and 21/100,000 in 2009. However, it is important to note that only about 30% of coccidioidomycosis cases reported to the ADHS contain information about race and only about 20% contain information about ethnicity, so these numbers may be unreliable.

The highest rates of coccidioidomycosis cases continue to occur in Arizona’s most populated counties of Maricopa, Pima, and Pimal, which experience arid to semiarid climates with mild winters and hot summers. Maricopa had rates of 97/100,000 from 2006–2008 and 204/100,000 in 2009. Pima had rates of 87/100,000 from 2006–2008 and 130/100,000 in 2009. Pinal had rates of 83/100,000 from 2006–2008 and 155/100,000 in 2009. These rates are markedly different than rates in counties at higher elevations, such as Apache and Coconino (both counties had rates of seven cases per 100,000 population from 2006–2008; and 14 and 18 cases per 100,000 population in 2009, respectively). Interestingly, Yuma County, an area with similar climate conditions to Maricopa, Pima and Pinal, had low rates of coccidioidomycosis (seven cases per 100,000 population from 2006–2008 and six cases per 100,000 population in 2009).

Arizona continues to have high numbers of coccidioidomycosis cases. Comparing 2009 to previous years’ data, the influx of positive EIA reports of coccidioidomycosis has coincided with a decrease in the median age of coccidioidomycosis cases as well as a shift in the majority of cases being female rather than male. Although EIA testing is the easiest and least expensive to perform, is widely used and provides a faster turnaround time, its sensitivity and specificity are not fully established [17,18]. The ADHS is carrying out further investigations in understanding EIA sensitivity and specificity in order to improve surveillance of coccidioidomycosis in Arizona.

### 2.2. Monitoring Laboratory Reporting of Coccidioidomycosis

The Council of State and Territorial Epidemiologist (CSTE) and the Centers for Disease Control and Prevention (CDC) require laboratory and clinical criteria to meet the case definition for coccidioidomycosis. The laboratory criteria consist of positive culture, histopathologic or molecular evidence; or immunologic evidence in the form of detection of IgM or IgG by immunodiffusion (ID), enzyme immunoassay (EIA), latex agglutination, tube precipitin or complement fixation. Clinical criteria require influenza-like signs and symptoms; pneumonia or other pulmonary lesion; erythema nodosum or erythema multiforme rash; involvement of bones, joints, or skin by dissemination; meningitis; or involvement of viscera and lymph nodes. Because of Arizona’s large number of cases and limited resources, ADHS utilizes only the laboratory component of the CSTE coccidioidomycosis case definition.

In 2007, two major laboratories (Laboratory A and Laboratory B) accounted for 46% of all coccidioidomycosis cases reported to the state health department. In order to validate public health reporting of coccidioidomycosis, data on positive coccidioidomycosis tests were obtained retrospectively from the two laboratories for the period of March through May 2008, independent of routine reporting. During the validation period, Laboratory A used EIA as a screening tool and routinely only reported positive coccidioidomycosis results that were confirmed by either immunodiffusion or complement fixation. Laboratory B reported any positive results from immunodiffusion, complement fixation or EIA testing. These positive tests underwent three rounds of validation: the results were first compared to Arizona’s surveillance database, which tracks only incidence reporting and not repeat or chronic testing, using an exact match on first and last name and birth date; this database contains coccidioidomycosis cases reported since January 2006. Second, unmatched results were compared to a historical database, which contains reports from 1998 through 2005, using the same matching criteria of first and last name and date of birth. This step was conducted since coccidioidomycosis cases are only added to Arizona’s database once, regardless of the number of tests conducted; a 2008 test that matches a 2004 case was presumably not entered in 2008 because it was received in the earlier year. Finally, any remaining unmatched results were hand-matched to both databases to identify matches with possible misspellings or missing information.

This validation showed that 651 (98%) of 664 patients with records provided to the state health department in the validation data matched for Laboratory A. Laboratory B matched for 427 (85%) of 517 patients with positive tests reported. Missing reports were more likely to be EIA tests than complement fixation or immunodiffusion. These findings provide validation that most coccidioidomycosis tests are being reported to the state health department from these two large laboratories.

### 2.3. Coccidioidomycosis as a Cause of Community-Acquired Pneumonia

The clinical syndrome of coccidioidomycosis is very similar to other respiratory diseases, and difficult to recognize for both the providers and the public. A small study in Pima County established that 16 (29%) of 55 cases of community-acquired pneumonia (CAP) had positive serological tests for coccidioidomycosis, and that symptoms were insufficient to distinguish coccidioidomycosis from other pneumonias [19]. Another small study in Maricopa County found that 6 (17%) of 35 CAP patients with paired testing had coccidioidomycosis [20]. However, testing of CAP patients has been shown to be performed infrequently. A study at two healthcare systems in Maricopa County found that only 2% and 13% of ambulatory CAP patients were serologically tested for coccidioidomycosis [21]. In 2006, ADHS made a strong recommendation to test patients with symptoms of CAP, based on the knowledge that in areas where *Coccidioides* spp. are endemic coccidioidomycosis represents a substantial underlying cause of CAP.

In order to further evaluate this recommendation, ADHS conducted an investigation at two large sentinel hospitals in Pima County. All patients with a diagnosis of pneumonia admitted to the emergency departments from these two hospitals from 2007–2008 were screened to determine the proportion tested for coccidioidomycosis. Enhanced chart review was used to determine those that had community-acquired pneumonia. A stricter definition for community-acquired pneumonia was used for enrollment than in previous studies [19]. Patients were classified as having CAP if they had the International Classification of Diseases, 9th revision codes beginning with 480–487 (included all types of pneumonia and influenza), radiographic evidence of a new infiltrate documented, at least one respiratory sign or symptom, and reported or observed fever or chills. The proportion of CAP patients receiving a test for coccidioidomycosis was below 10%, but rose to over 40% at the end of the first year. Targeted education to emergency room physicians at these hospitals may have increased the testing of patients. Physician educational efforts included presentations by an ADHS infectious disease physician and an educational poster prompting physicians to test CAP patients for coccidioidomycosis. In addition, in collaboration with the University of Arizona’s Valley Fever Center for Excellence (VFCE), ADHS developed a continuing medical education (CME) course, designed brochures and released a public service announcement encouraging patients to request that their doctors test them for coccidioidomycosis if they are experiencing cough, fever, and/or exhaustion. Furthermore, ADHS is currently conducting surveillance of severe acute respiratory infections and will be testing patients for coccidioidomycosis among other etiologies.

The underdiagnosis and misdiagnosis of coccidioidomycosis represent a public health concern as patients with this disease often incur weeks to months of disability and time away from work. These illnesses also utilize significant amounts of medical resources, including hospitalizations, resulting in a tremendous economic burden (detailed more fully, below). As a result, there needs to be increased attention for seeking diagnosis earlier in patients with CAP and endemic exposure to *Coccidioides* spp., as recommended in current practice guidelines [22]. In addition to providing a patient with an exact diagnosis, other potential benefits would be decreasing unneeded and ineffective treatment with antibacterial drugs, reducing the need for many other diagnostic tests and procedures, and the possibility of identifying earlier the infrequent but serious complication of hematogenous spread beyond the chest, resulting in disseminated sites of infection that are more difficult and expensive to treat.

### 2.4. Enhanced Surveillance of Coccidioidomycosis

In 2007, ADHS initiated enhanced surveillance of coccidioidomycosis to characterize the effects of this disease on Arizona’s population, healthcare system, and economy and to validate its use of the laboratory-exclusive coccidioidomycosis case definition. Every tenth patient with coccidioidomycosis that was reported to the state health department was interviewed using a standardized questionnaire. Interviewees were asked questions regarding signs and symptoms of coccidioidomycosis, healthcare-seeking behaviours and treatment, whether they had known about coccidioidomycosis before their diagnoses, and how the disease affected their daily lives. A total of 493 (9%) people were interviewed out of 5,664 coccidioidomycosis cases reported from January 2007 through February 2008. The results of the study have been reported previously [23].

This investigation found that persons with coccidioidomycosis waited a median of 11 days before they sought healthcare for their symptoms, and it took a median of 23 days from the day they sought healthcare to the day that coccidioidomycosis was diagnosed. Patients reported a median of two visits to a healthcare provider before a *Coccidioides* test was ordered. Over one-fourth (26%) visited a healthcare provider more than 10 times for their illness. Almost half (44%) of coccidioidomycosis patients visited an emergency department during the course of their illness. Over 40% were hospitalized overnight for their illness. Hospital charges totaled $86 million among Arizonans who had primary or secondary diagnoses of coccidioidomycosis in 2007 (median charge of $30,000 and mean charge of $49,000 per visit).

Notably, persons with coccidioidomycosis experienced symptoms for a median of 120 days. Out of the 493 patients interviewed, 225 (46%) were employed at the time of illness. Out of those employed, 167 (74%) missed a median of 14 days from work. Daily activities could not be performed for a median of 47 days for 369 (75%) patients.

Persons with coccidioidomycosis who knew about the disease before seeking healthcare were more likely to get an earlier diagnosis of the disease than those who did not know about the disease (median 20 days *vs.* 25 days, p = 0.04) and were also twice as likely to ask their healthcare providers to test them for coccidioidomycosis (95% confidence interval (CI) 1.0–3.2, p = 0.05). Furthermore, Caucasian patients of were more likely to know about coccidioidomycosis before their diagnoses than were patients of other racial groups (95% CI 1.7–4.3, p < 0.01).

Antifungal treatment for coccidioidomycosis was prescribed for 303 (61%) of patients. About 60% (289) were treated with antibacterial agents. Of those, 92 (32%) received more than one course of antibacterial drugs. Of the 493 patients, 469 (95%) met the CSTE clinical case definition. Thirteen (3%) reported no symptoms.

The enhanced surveillance investigation revealed substantial personal and economic burdens due to coccidioidomycosis among Arizonans with respect to delays in diagnosis, duration and severity of illness, and healthcare utilization and costs. This investigation also highlighted the importance of public and provider education in reducing delays in diagnosis and thereby minimizing unnecessary use of antimicrobial drugs, relieving patient anxiety, and enabling early recognition and treatment of this disease. These findings also support the use of a laboratory-exclusive case definition in coccidioidomycosis-endemic areas for surveillance purposes to reduce resources needed for an accurate assessment of the extent of the disease and its effects.

### 2.5. Conclusions

In summary, Arizona surveillance data demonstrate a high burden of disease due to coccidioidomycosis in the state, and the surveillance system accurately reflects coccidioidomycosis reports from laboratories. With the recent changes in coccidioidomycosis reporting by a major laboratory and perhaps other laboratories and hospital facilities, further analysis should be done to characterize this disease. Educational efforts in testing patients with CAP have increased awareness and diagnosis of coccidioidomycosis. Enhanced surveillance highlights the significant symptom duration and delays in diagnosis of this disease and also further supports the need for physician and provider education in reducing these delays for optimal patient care and treatment. Because of the huge effects of this disease upon Arizona’s residents, healthcare system and economy, continued surveillance, research, and education of this disease is necessary.

## 3. The Public Health Impact of Coccidioidomycosis in California, 2001–2009

To describe the epidemiology of coccidioidomycosis in California during 2001–2009, cases of coccidioidomycosis reported to the California Department of Public Health (CDPH) between 2001 and 2009 and cases of hospitalized coccidioidomycosis included in the California Patient Discharge Data Set 2001–2008 were reviewed.

### 3.1. Materials and Methods

The California Department of Public Health (CDPH) utilizes a passive reporting surveillance system for coccidioidomycosis cases and outbreaks. Health care providers and, most recently, since 2010 laboratories are mandated by the California Code of Regulations, Title 17, to report cases of coccidioidomycosis to their local health departments (LHDs) that, in turn, report these cases to CDPH [24]. The case definition for surveillance reporting is based on clinical criteria and laboratory evidence of infection defined by the Council of State and Territorial Epidemiologists (CSTE) and the federal Centers for Disease Control and Prevention (CDC) [25].

We analyzed the CDPH surveillance data on coccidioidomycosis cases with an estimated date of illness onset from 2001 through 2009. The estimated onset date for each case was defined as the date closest to the time when symptoms first appeared, ranging from the first appearance of symptoms to the date the report was made to CDPH. A probabilistic de-duplication algorithm was used to include only the first report of the disease per person during the study period. Additionally, the provisional reported data (as of second week of January 2011) of cases with estimated illness onset date of 2010 were de-duplicated and reviewed. Publications of outbreaks or locally increased cases in special populations during the study period were briefly discussed.

The coccidioidomycosis hospitalization data for the period 2001–2008 were extracted from the California Patient Discharge Data Set. The data set included inpatient discharge diagnosis from all California nonfederal hospitals. The data were probabilistically de-duplicated to include first hospitalized cases during the study period. Cases with codes for coccidioidomycosis (114.0–114.5 and 114.9) from the International Classification of Diseases, Ninth Edition, were selected.

We calculated state and county-specific case rates by the estimated onset year as well as the yearly hospitalization rates per 100,000 California population. To calculate rates, we used projections for state and county specific population totals that were published by the State of California, Department of Finance [26]. Rates were defined as unreliable if their coefficient of variations (relative standard errors) were 23 percent or more (a threshold recommended by the National Center for Health Statistics). Analyses were conducted using SAS Release 9.1 (SAS Institute, Inc, Cary North Carolina). We examined temporal trends by plotting incidence cases (de-duplicated to account for previous reports on a given subject) and rates, first hospitalization cases and rates, and percent hospitalization over time. Spatial trends of cases were depicted by mapping county-specific average incidence rates for years 2001–2004, 2005–2008, and 2009 using ArcGIS version 9.3 (ESRI, Inc, Redlands, California). Unreliability of rates was marked on the maps using dash lines.

### 3.2. Results

**Figure 3.**
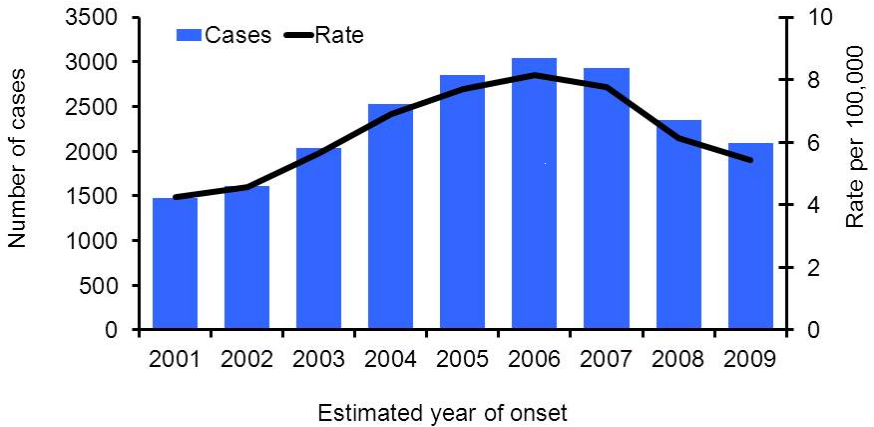
Coccidioidomycosis incidence cases and incidence rates in California, 2001–2009.

As of June 2010, CDPH received reports of 20,931 cases of coccidioidomycosis with estimated illness onset dates from 2001 through 2009, reaching a peak at 3,043 reported cases in 2006 (Figure 3). The annual incidence rate per 100,000 population increased by 91.1% from 4/100,000 in 2001 to 8/100,000 in 2006, and decreased by 33.4% subsequently to approximately 5/100,000 in 2009.

**Figure 4.**
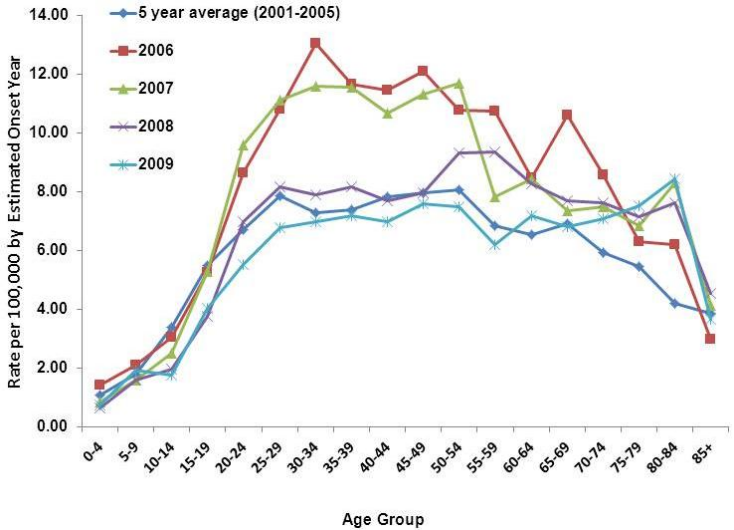
Coccidioidomycosis incidence rates by age-group in California, 2001–2009.

The majority of the 20,931 reported cases were male (65%). There were increasing trends in incidence rates in age groups 0–4 to 25–29 across all years (Figure 4). The incidence rates stayed relatively steady in age groups 25–29 to 50–54, and declined with some fluctuations in age groups 55–59 and older. The incidence rate was highest among those 30–34 years old in the peak year 2006 (13 per 100,000).

**Figure 5.**
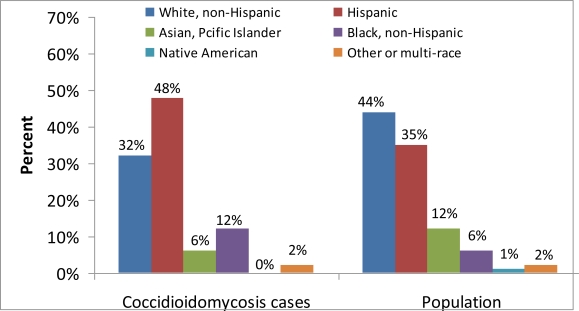
Coccidioidomycosis incidence cases and population by reported race/ethnicity in California, 2001–2009.

**Figure 6.**
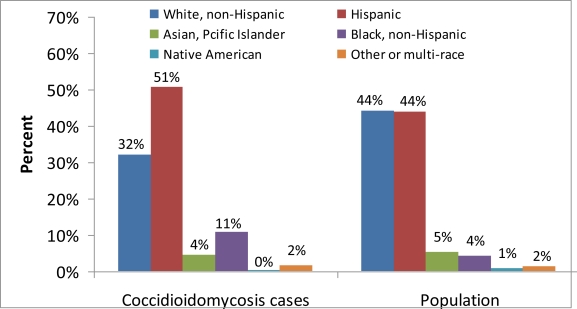
Coccidioidomycosis incidence cases and population by reported race/ethnicity in California *Coccidioides*-endemic areas (Fresno, Kern, Kings, Madera, San Luis Obispo, and Tulare), 2001–2009.

Cases with complete race/ethnicity data reported Hispanic and Black, non-Hispanic races/ethnicities more frequently than would be expected based on the overall demographic profile of California (Figure 5), as well as the most highly-endemic areas of California (Fresno, Kern, Kings, Madera, San Luis Obispo, and Tulare) (Figure 6). Thirty-five percent of the records in California and fifty percent of the records in the California *Coccidioides*-endemic areas were missing race/ethnicity information; therefore, incidence rates were not calculated.

**Figure 7.**
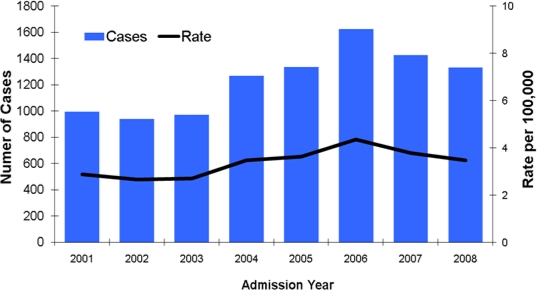
Coccidioidomycosis first hospitalization cases and rates in California, 2001–2008.

There were 9,898 unique hospitalized cases from 2001 through 2008. The highest number of cases and hospitalization rates were in 2006 (1,626 cases; 4 per 100,000) (Figure 7).

**Figure 8.**
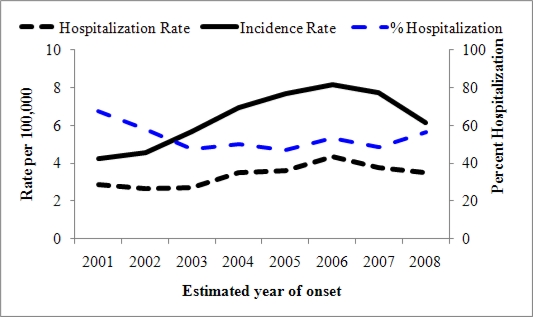
Coccidioidomycosis incidence and first hospitalization rates and percent hospitalization in California, 2001–2008.

Figure 8 presents coccidioidomycosis incidence and first hospitalization rates per 100,000 population as well as percent hospitalization in California. Reported cases and hospitalization rates followed similar trends over time. The percent hospitalization (hospitalization rate by reported incidence rate) decreased in 2003, followed by a plateau from 2003 to 2007, and increased in 2008.

**Figure 9.**
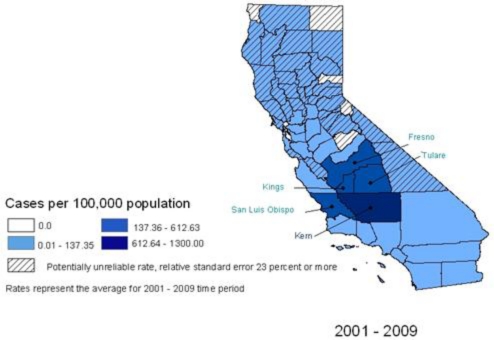
Coccidioidomycosis county-specific incidence rate ranges, California, 2001–2009.

The counties of Kern (annual average rate: 144/100,000; cumulative cases: 9,998), Kings (annual average rate: 68/100,000; cumulative cases: 900), Tulare (annual average rate: 37/100,000; cumulative cases: 1,374), Fresno (annual average rate: 33/100,000; cumulative cases: 2,635), Madera (annual average rate: 15/100,000; cumulative cases: 196), and San Luis Obispo (annual average rate: 31/100,000; cumulative cases: 734) had the highest annual average incidence rates during the study period. These counties are established as being *Coccidioides*-endemic areas and their cumulative cases represent 76% of all cases reported during the study period. There were only five out of the 58 counties in California with no reported cases during the entire study period. Figure 9 presents county-specific cumulative incidence ranges per 100,000 population for years 2001–2009 across California. Counties with unreliable rates within the time period were marked with hatched lines.

The study sought to include the most recently reported data by examining the 2010 provisional data, particularly since coccidioidomycosis became laboratory-reportable in California in 2010. As of the second week of January 2011, 3,461 (9 per 100,000 population) provisional cases of coccidioidomycosis with estimated illness onset dates in 2010 have been reported to CDPH.

Regarding focal outbreaks or clusters of infections, during the study period, there were two reported outbreaks in California, one in 2001 among United States Navy SEALS training in an endemic area, and one in 2007 among civilian construction workers excavating soil at a military base. In the Navy SEALs outbreak, ten (45%) of 22 men had serologic evidence of recent *Coccidioides* infection and five (50%) of the ten had abnormal chest radiographs [27]. In the construction workers outbreak, eight (67%) of 12 men had serological evidence of infection and seven had abnormal chest radiographs [28]. During 2002–2006, a naval air station in the California Central Valley reported increased rate of coccidioidomycosis among active duty personnel with increased disseminated cases to a high of 30% of diagnosed cases by 2006 [29]. And substantially increased numbers of cases have been reported among inmates of California State prisons situated in the endemic area [30,31].

### 3.3. Discussion, Limitations, and Comments

During 2001–2009, the annual number of incidence cases and rates of reported coccidioidomycosis in California increased steadily to a peak in 2006, and then decreased. The annual number of incidence cases and rates during this period were higher than the annual numbers and rates between 1995 and 2000 (data not shown). The annual number and rate of coccidioidomycosis hospitalizations have followed the trend of reported cases during this time, peaking in 2006 and then decreasing.

The distribution of reported cases by counties in California reflected the importance of the endemic San Joaquin Valley and San Luis Obispo in the Central Coast, but cases have been reported from all except five counties during this time. Inmates of California State prisons situated in the endemic areas contributed to the incidence of coccidioidomycosis in the *Coccidioides*-endemic areas. Further studies are needed to describe the demographic and risk factors of inmate *vs.* non-inmate cases in California. The increase in the number of provisional 2010 incidence data compared to 2009 could partly be due to the impact of making the disease laboratory-reportable in California. However, the 2010 data are provisional at this point and may still be subject to reporting delays. Future studies are needed to assess any possible impact of the 2010 change in regulation on timeliness and completeness of the surveillance data. Collectively, the data support the conclusion that coccidioidomycosis continues to be an important public health problem and burden in California

## 4. Diagnosis and Treatment of Coccidioidomycosis

The spectrum of coccidioidomycosis was very well appreciated long before the first effective antifungal drug, amphotericin B, was available as a treatment. For example, a monograph on coccidioidomycosis, which is quite true to our current understanding of disease manifestation, was published in 1958 [32], just about the time amphotericin B was first coming into use [33]. It is quite clear that most patients who become ill exhibit findings of a respiratory tract infection and that, whether treated or not, most infections resolve. When amphotericin B was the only treatment available, its toxicity, untoward reactions, and its parenteral route of administration resulted in recommendations for its use only in patients with clear indication of progressive, coccidioidal infections and not in patients with the self-limited form of disease, commonly known as Valley Fever.

Because most coccidioidal infections are self-limited, many clinicians discount the value of diagnosing the initial coccidioidal pneumonia, even within areas highly endemic for coccidioidomycosis, and do not routinely test patients with community-acquired pneumonia (CAP) for coccidioidomycosis even though the likelihood of this cause approaches 30% [19,20]. As mentioned above, a study of approximately 150 outpatients in Phoenix, Arizona, found that only 2% to 13% of patients with community acquired pneumonia in a one-year period were tested serologically for coccidioidomycosis [21]. However, what has re-emerged in the recent surveillance activities in Arizona [23], is that even for patients with the self-limited pulmonary syndrome the impact of this disease is often substantial. As detailed elsewhere in this review, patients with coccidioidomycosis often incur weeks to months of disability and time away from work. These illnesses also utilize significant amounts of medical resources, including hospitalization. These findings are consistent with the descriptions published seventy years earlier [34,35] that are likely not familiar to most clinicians. As a result, there needs to be increased attention for seeking diagnosis earlier in patients with CAP and possible endemic exposure to *Coccidioides* spp., as is the recommendation of current practice guidelines [22]. In addition to providing a patient with an exact diagnosis, in itself very valuable, other potential benefits would be reducing unneeded and ineffective treatment with antibacterial drugs, reducing the need for many other diagnostic tests and procedures, and the possibility of identifying earlier the infrequent but serious complication of hematogenous spread beyond the chest, resulting in disseminated sites of infection that are more difficult and expensive to treat.

### 4.1. Improving Diagnostics for Early Coccidioidal Infections

The increased interest in earlier diagnosis of coccidioidal infections has brought into focus the limitations of current diagnostic tests for coccidioidomycosis. Although isolating *Coccidioides* spp. in culture results in a definitive diagnosis, fungal cultures, especially in the outpatient setting, are infrequently obtained in patients with CAP. Culture is more sensitive than direct examination of bronchoscopic specimens [36], and presumably would be very sensitive in the diagnosis of active coccidioidal pneumonia. However, the sensitivity of sputum cultures is not certain and the ability to obtain sputum from patients with coccidioidal pneumonia is often challenging since their coughing is frequently described as “non-productive.” Even when fungal cultures of sputum are submitted, there is frequently a delay of one or more weeks before a positive result is returned. Skin tests with coccidioidin or spherulin were widely used as a diagnostic for many decades, until being withdrawn from the market in the 1980’s (although a commercial entity is currently seeking FDA approval to re-launch spherulin in the USA). As a result of these limitations, a large majority of patients with suspected coccidioidal CAP are tested serologically, instead. Conventional immunodiffusion tests for anti-coccidioidal antibodies were derived from extensive experience spanning several decades [37] and provides strong evidence that positive tests are often indicative of recent or active infection. However, their sensitivity for detecting diagnostic antibodies in the first few weeks of illness is not nearly as great as later in infection or in patients with chronic disease. In one report, conventional test results were non-diagnostic in first serum specimens from one-third to two-thirds of patients studied [18].

Increasing the sensitivity of diagnostic tests for early coccidioidal infection could potentially be accomplished by any of several approaches. One would be to improve serologic testing itself. For example, a commercially available enzyme-linked immunoassay kit, Premier EIA, (Meridian Diagnostics, Inc., Cincinnati, OH) may be more sensitive than the conventional immunodiffusion tests [38]. However, the documentation for its performance is scant and the proprietary antigen that the test uses to detect anti-coccidioidal antibodies has not been publically disclosed.

Another approach would be to use nucleic-acid amplification technologies to detect *Coccidioides*-specific sequences in clinical specimens. In an early report, a PCR-based assay demonstrated that such sequences were present in patients [39], and more recent reports have shown good correlation between a real-time PCR method and culture results from bronchoalveolar lavage fluids [40] and the ability to detect coccidioidal DNA before the appearance of diagnostic antibodies in patients [41]. At least one medical center has systematically employed an experimental PCR method in suspected cases, targeting the ITS2 region of the fungal genome, and has reported results compared to traditional culture methods [42]. While showing promise as a more rapid test, it is not clear that the method can supplant more traditional diagnostic methods.

Detecting coccidioidal antigens in clinical specimens might also improve early detection of coccidioidomycosis. Several research reports have suggested that circulating coccidioidal antigens are present during naturally-acquired human infections [43–45]. In one report, antigenemia appeared to precede the detection of antibodies [18], and others have demonstrated polysaccharide antigenuria and antigenemia in patients with very extensive coccidioidal infections [46,47].

### 4.2. Current and Future Therapies for Coccidioidomycosis

Amphotericin B was once the only effective treatment for coccidioidomycosis [48], and is still considered an effective, perhaps the most effective antifungal agent [49]. In vitro, amphotericin B exerts its inhibitory effort almost immediately and some clinicians believe that clinical response is fastest with this drug as compared to newer agents. A major drawback to using amphotericin B is that it requires parenteral administration, which makes long-term treatment more cumbersome. As a therapy for coccidioidal meningitis, intravenous therapy with the original formulation of amphotericin B was not effective. While experimental evidence raises the possibility that some of the lipid formulations administered intravenously may be better [50,51], this has not yet been corroborated with clinical experience. As a result, when amphotericin B is used to treat coccidioidal meningitis, it requires administration directly into the CSF [52,53], which adds additional risk and morbidity to its use. Another major difficulty with amphotericin B therapy is that it is very toxic; fever, nausea, and myalgia are frequent in association with administration. Dose-related toxicity commonly involves major organs and cumulative toxicity may result in permanent renal failure, requiring dialysis or organ transplantation.

Fortunately, over the past three decades, therapy for coccidioidal infections has improved [54]. Miconazole was the first alternative found to be effective against coccidioidal infections, demonstrating the potential for the azole class of antifungals [55]. Subsequently, orally-effective congeners, first ketoconazole [56,57] and later fluconazole and itraconazole [58], have largely replaced amphotericin B as first-line treatment. Although fluconazole and itraconazole are similar in their effectiveness, itraconazole may be more effective for skeletal lesions. Their relative safety and ease of administration has allowed treatment regimens to be extended for years and, in some patients, for life-long courses of treatment. Azole therapy has been particularly dramatic for the treatment of coccidioidal meningitis, which previously required intrathecal amphotericin B administration [59–61]. The newer azoles voriconazole and posaconazole have been approved by the FDA as treatment for some systemic mycoses and the relatively small amount of information available about their use in treating coccidioidomycosis suggests they may also be useful in treating this disease [62–66].

With these advances, there remain several limitations of current treatments. A notable gap in our understanding in managing coccidioidal infections is the complete absence of randomized trials of any azole antifungal therapy as a treatment of early coccidioidal infections. A prospective observational study at one Veterans Administration medical center compared the outcome of patients with early coccidioidal pneumonia who were or were not treated with an oral azole (usually fluconazole) [67]. There was no evident difference between the two groups in their rates of improvement. Moreover, complications of infection only occurred in the group of patients treated with antifungals after their treatment was discontinued. In other studies where subjects were treated because of chronic pulmonary or extrapulmonary infections, azole treatment did not always result in measurable improvement, with treatment failure occurring in approximately 20% to 40% of cases [58,62]. Of patients who did improve during treatment, many relapsed even after protracted courses of treatment were completed. These findings have led to the recommendation that for patients with the most serious manifestations of coccidioidomycosis, including all patients with coccidioidal meningitis [41], treatment be continued indefinitely. Collectively, these results underscore the need for new classes of therapeutics that can result in cures rather than just amelioration of disease.

Nikkomycin Z, a new antifungal drug which targets the cell wall of chitin-rich fungi, is currently in development for treating coccidioidomycosis. Nikkomycin Z, originally discovered in the 1970s, showed striking therapeutic effects when administered orally to mice infected with *Coccidioides* spp. [68]. Development of nikkomycin Z as a treatment for systemic mycoses was begun in the 1990’s but the sponsoring company went out of business and, without a commercial sponsor, the development program stalled. In 2005, the inactive IND for nikkomycin Z was transferred to the University of Arizona, which activated the program and completed a phase I multidose study [69]. A company, Valley Fever Solutions, has been formed primarily to assist the University of Arizona in developing the drug’s development [70]. Current efforts are focused upon manufacturing more nikkomycin Z which will be used in phase II clinical trials. It is hoped that with continued development, clinical confirmation of the preclinical data will drive the commercialization of this compound, offering an attractive therapeutic option in the treatment of coccidioidomycosis.

## 5. A Preventive Vaccine for Coccidioidomycosis

Based on epidemiologic and preclinical animal studies that date back to the 1940’s and 50’s, it had been established that persons who recovered from coccidioidal infection had durable immunity to re-infection that was, in the absence of profound immune suppression, life-long [71]. A large body of clinical and preclinical data that followed solidified this finding, supporting the possibility that a preventive vaccine could be created, which, if utilized, could impact the incidence and/or the severity of disease and the associated public health burden and economic consequences [72–74].

Trials in humans utilizing formalin-killed vaccine preparations of both the mycelial and spherule phases of the dimorphic fungus have been conducted, but uniformly resulted in local and systemic reactogenicity that limited the amount of the experimental vaccines that could be administered, with the net effect that the doses proved insufficient to trigger a uniformly sufficient immune response [75,76].

Because of the unacceptable toxicity of the killed whole-cell vaccine preparations, more recent efforts to create an effective vaccine have primarily focused on use of recombinant or native soluble protein formulations or live attenuated vaccines [77–84]. However, the challenges in discovering and developing an effective, preventive vaccine for coccidioidomycosis are daunting. The understanding of innate immunity and the basis for eliciting a protective immune response to coccidioidal infection continues to evolve. Many of the foundational immune components critical to protection are known; infection triggers a Class II-restricted T helper 1 (Th1) cellular immune response that is mediated, in part, by macrophages activated by IFN-γ and IL-12, which have been shown to be crucial in locally controlling infection [7,85–87]. Newer data also suggest a role for IL-17 [88]. Additionally, while earlier dogma held that antibodies were not a part of a protective immune response (with titers of IgG to coccidioidal antigens actually proportional to the severity of disease), more current studies have ascribed a role for humoral immunity, particularly in the early phases of disease [89].

Transient duration of protection induced by protein-based coccidioidal vaccines has been an issue, with some experimental vaccines that initially provide protection against lethal challenge later failed to prevent recurrence of progressive, fatal disease in mice [90,91]. In addition, adjuvants that are appropriate to help elicit a robust Th1 response have not yet met a safety profile in humans that is suitable for widespread use in a vaccine for what is largely a benign disease.

The cost-effectiveness of a coccidioidal vaccine was projected in one study. Vaccination amongst various age groups in the endemic area was predicted to be cost saving among infants, teens, and resident and immigrant adults, but was economically unfavorable among resident seniors and among immigrant seniors. [92]. As one metric, based on the assumptions and models detailed in that study, vaccination for coccidioidomycosis in the endemic area was predicted to save 0.03 to 0.4 life days per person vaccinated over no vaccination among various populations. In comparison, infant vaccination against measles, mumps, rubella, and pertussis each save 2.7, 3, 0.3 and 3.3 life days, respectively [93]. However, an assessment of the cost-effectiveness of a coccidioidal vaccine should also take into account the prolonged morbidity, the number of work days and quality of life lost, and the familial and societal burdens of this disease; criteria that have particular importance given the increasing incidence of this disease, with millions of people living in or moving to the endemic areas.

## 6. Discussion

We found that the incidence of coccidioidomycosis has dramatically increased over the past decade, and that the case and total economic costs associated with acquisition of symptomatic disease have substantially increased since the vaccine cost-effectiveness report was published in 2001. Despite enhanced reporting requirements instituted by both Arizona and California, greater physician and provider education leading to more stringent surveillance and timely use of diagnostics is needed to reduce delays in diagnosis and the provision of optimal patient care and treatment. Because of the economic costs attributable to this disease, the burden on the private, public, and prison healthcare infrastructure, the toll of morbidity on the afflicted and their families, and the lack of curative therapeutics, the need for improved therapeutics and a preventive vaccine for coccidioidomycosis have never been greater.
